# Molecular dissection of human b-cell tolerance - insights from primary immunodeficiencies

**DOI:** 10.1186/1546-0096-12-S1-P40

**Published:** 2014-09-17

**Authors:** Henner Morbach, Greta Meyers, Yen-Shing Ng, Jean-Nicolas Schickel, Laurence Menard, Sergei Rudchenko, Jessica Rojas, Charlotte Cunningham-Rundles, Mary Conley, Ismail Reisli, Jose Franco, Eric Meffre

**Affiliations:** 1 Department of Immunobiology, Yale University, New Haven, USA; 2 Pediatric Rheumatology and Immunology, University Children's Hospital, Würzburg, USA; 3 Laboratory for Biochemistry and Molecular Immunology, Hospital for Special Surgery, New York, USA; 4 Group of Primary Immunodeficiencies, University of Antioquia, Medellin, Colombia; 5 Department of Medicine, Mount Sinai Medical Center, New York, USA; 6 Department of Pediatrics, University of Tennessee, Memphis, USA; 7 Department of Immunology and Allergy, Meram Medical Faculty, Selcuk University, Konya, Turkey; 8 Group of Primary Immunodeiciencies, University of Antioquia, Medellin, Colombia

## Introduction

B cells play a central role in the pathogenesis of many autoimmune diseases. Therefore, understanding the mechanisms that regulate B-cell tolerance in humans is important for the development of new therapeutic strategies. Patients with monogenic diseases provide rare opportunities to study the impact of specific gene mutations on the regulation of human B cell tolerance. By this, we could show that alterations in B-cell receptor (BCR) and Toll-like receptor (TLR) signaling pathways result in defective central B-cell tolerance.

## Objectives

The aim was to further dissect the BCR and TLR signaling pathways involved in the establishement of central B-cell tolerance in humans. In detail, we aimed to analyze the role of CD19 in mediating central B-cell tolerance in humans. CD19 is a co-receptor expressed on B cells and is involved in the amplification of B-cell responses; its expression is decreased in patients with systemic lupus erythematosus (SLE), suggesting that proper CD19 expression may normally prevent autoimmunity. Additionally, CD19-deficient patients suffering from an antibody deficiency are also prone to develop systemic autoimmune diseases resembling SLE.

## Methods

To test the function of the central B-cell tolerance checkpoint in humans, we analyzed by ELISA and immunofluorescence tests the reactivity of recombinant antibodies cloned from single transitional B cells from individuals carrying CD19 mutations. Additionally, we analyzed alterations in TLR and BCR signaling pathways in CD19-deficient human B cells using flow cytometry and immunoblotting.

## Results

We found that individuals carrying CD19 mutations displayed defective central B-cell tolerance checkpoints. In addition, CD19-deficient transitional B cells were enriched in anti-nuclear clones, a feature previously observed in IRAK4- and MYD88-deficient patients in which TLR7/9 sensing nucleic acids cannot signal. Therefore, we investigated the functions of these TLRs in B cells in the absence of CD19 expression. CD19-deficient human B cells displayed defective up-regulation of activation markers after TLR7/9 triggering and failed to induce BTK, AKT but not p38 MAPK or Iκ-Bα phosphorylation after TLR7/9 stimulation. Additionally, inhibitors blocking BTK, AKT and PI3K function as well as mutations in BTK impaired CD19-dependent TLR7/9 responses in healthy donor’s B cells.

## Conclusion

Thus, CD19 and its PI3K/BTK/AKT signaling pathway is essential for B-cell activation and the establishment of central human B-cell tolerance by mediating the function of both BCRs and TLRs that recognize self-antigens during early B-cell development

## Disclosure of interest

None declared.

